# A randomized, placebo-controlled, double-blind, prospective trial to evaluate the effect of vildagliptin in new-onset diabetes mellitus after kidney transplantation

**DOI:** 10.1186/1745-6215-11-91

**Published:** 2010-10-06

**Authors:** Michael Haidinger, Johannes Werzowa, Hans-Christian Voigt, Johannes Pleiner, Gunar Stemer, Manfred Hecking, Dominik Döller, Walter H Hörl, Thomas Weichhart, Marcus D Säemann

**Affiliations:** 1 Clinical Division of Nephrology and Dialysis, Department of Internal Medicine III, Medical University of Vienna, Austria; 2 Koordinierungszentrum Klinische Studien (KKS), Medical University of Vienna, Austria; 3 Pharmacy Department, Vienna General Hospital, Austria

## Abstract

**Background:**

New-onset diabetes mellitus after transplantation (NODAT), a frequent and serious complication after transplantation, is associated with decreased graft and patient survival. Currently, it is diagnosed and treated primarily according to existing guidelines for type II diabetes. To date, only a few trials have studied antidiabetic drugs in patients with NODAT. Vildagliptin is a novel dipeptidyl peptidase-4 (DPP-4) inhibitor that improves pancreatic islet function by enhancing both α- and β-cell responsiveness to increased blood glucose. Experimental data show potential protective effects of DPP-4 inhibitors on islet function after exogenous stress stimuli including immunosuppressants. Therefore, the therapy of NODAT with this class of compounds seems attractive. At present, vildagliptin is used to treat type II diabetes as monotherapy or in combination with other antidiabetic drugs, since that it efficiently decreases glycated hemoglobin (HbA1c) values. Additionally, vildagliptin has been shown to be safe in patients with moderately impaired kidney function. This study will evaluate the safety and efficacy of vildagliptin monotherapy in renal transplant recipients with recently diagnosed NODAT.

**Methods/Design:**

This study is a randomized, placebo-controlled, double-blind, prospective phase II trial. Using the results of routinely performed oral glucose tolerance tests (OGTT) in stable renal transplant patients at our center, we will recruit patients without a history of diabetes and a 2 h glucose value surpassing 200 mg/dl (11.1 mmol/l). They are randomized to receive either 50 mg vildagliptin or placebo once daily. A total of 32 patients with newly diagnosed NODAT will be included. The primary endpoint is the difference in the 2 h glucose value between baseline and the repeated OGTT performed 3 months after treatment start, compared between the vildagliptin- and the placebo-group. Secondary endpoints include changes in HbA1c and fasting plasma glucose (FPG). The safety of vildagliptin in renal transplant patients will be assessed by the number of symptomatic hypoglycemic episodes (glucose <72 mg/dl or 4 mmol/l), the number of adverse events, and possible medication-associated side-effects.

**Discussion:**

NODAT is a severe complication after kidney transplantation. Few trials have assessed the safety and efficacy of antidiabetic drugs for these patients. The purpose of this study is to assess the safety and efficacy of vildagliptin in renal transplant patients with NODAT.

**Trial Registration:**

ClinicalTrials.gov NCT00980356

## Background

New-onset diabetes after transplantation (NODAT), also called post-transplant diabetes mellitus (PTDM), remains a severe metabolic complication in patients after organ transplantation. NODAT leads to an increased incidence of cardiovascular disease (CVD) and consequently reduced graft and patient survival [1,2]. In non-transplanted patients, diabetes mellitus (DM) has been identified as a major independent risk factor for CVD [3]. CVD includes atherosclerotic coronary heart disease, heart failure, myocardial infarction, stroke and peripheral vascular disease [4]. Patients with CVD and DM suffer from a worse prognosis for survival than patients without these conditions. In organ transplant recipients, mortality due to CVD remains the most common cause of mortality [1]. In renal transplant recipients NODAT is associated not only with increased cardiovascular morbidity and mortality, but also with impaired long-term graft function and increased risk of graft loss [4,5]. Hence, NODAT needs medical attention and treatment and therefore clinical trials with antidiabetic drugs for the therapy of NODAT remain of high interest.

The reported incidence of NODAT varies between 2 and 53%. This high variability is due the lack of a standard definition in clinical studies [6]. Some reports define NODAT by the requirement for exogenous insulin without further examinations, such as an oral glucose tolerance test (OGTT). Currently, the diagnosis of NODAT is based on guidelines for type II diabetes (T2DM) from the American Diabetes Association (ADA), which include impaired glucose tolerance (IGT) and impaired fasting glucose (IFG) as diagnostic parameters [7]. Development of NODAT has modifiable (e.g. body weight, immunosuppressive drug therapy) and non-modifiable (e.g. age, ethnicity, polycystic kidney disease) risk factors [8]. The role of immunosuppressants (e.g. corticosteroids or calcineurin inhibitors (CNIs)) in the clinical course of diabetes is clearly established, and disease development is probably mediated by an increased beta-cell apoptosis and impaired insulin sensitivity [4,9]. The incidence of steroid-induced diabetes is related to the treatment duration and the dose of corticosteroids [10]. Some authors propose steroid reduction or complete withdrawal as a means to reduce the incidence of NODAT, but steroid withdraw has been associated with an increased risk for graft rejection [4].

Most centers currently follow so-called "step-up" strategies established for the treatment of T2DM starting with non-pharmacological therapies and life-style modification, subsequently followed by oral antidiabetic therapy and finally insulin [4]. Pharmacodynamic and pharmacokinetic drug properties may be altered in patients with renal impairment and new drugs have to be studied regarding safety and effectiveness in patients with impaired renal function. In renal transplant patients, drugs are at additional risk of interacting with immunosuppressive agents as well as with other co-medications [11].

Vildagliptin, a dipeptidyl peptidase IV (DPP-4) inhibitor that, belongs to a new class of oral antidiabetic drugs [12]. DPP-4 inhibitors enhance the activity of incretin hormones in response to a glucose load by blocking the hormones responsible for incretin degradation [13]. Incretins are gut hormones that are secreted from enteroendocrine cells into the blood within minutes after food intake. The incretin hormones *glucose-dependent insulinotropic polypeptide *(GIP) and *glucagon-like peptide-1 *(GLP-1) have been reported to exert numerous metabolic effects contributing to the regulation of blood glucose levels [14]. Vildagliptin decreases glycated hemoglobin (HbA1c) in patients with T2DM when given as monotherapy or combined with metformin or glitazones [15-19]. Furthermore, vildagliptin has been shown to be safe in patients with mild to moderately impaired kidney function [20].

This study aims to assess the safety and efficacy of vildagliptin in patients with NODAT.

## Methods/Design

### Hypothesis

Vildagliptin improves glucose metabolism in patients suffering from newly diagnosed NODAT.

### Objectives

This 16-week trial aims to evaluate the safety and efficacy of vildagliptin in stable renal transplant recipients with newly diagnosed NODAT.

The primary outcome parameter will be the difference in 2 h glucose levels obtained during an OGTT between stable renal transplant patients receiving vildagliptin or placebo after 3 months treatment.

The secondary study outcomes will include change in HbA1c and fasting plasma glucose after three months of treatment, the safety of vildagliptin in renal transplant recipients regarding kidney function, liver function and the potential for drug-drug interactions with immunosuppressive medications (Intention to treat (ITT) analysis), the safety of vildagliptin for glycemic control in patients with impaired kidney function, and the long-lasting effects of vildagliptin on β-cell function one month after treatment stop.

### Study design and setting

This study is a prospective, single-center, double-blind, randomized, placebo-controlled, phase II trial in patients with newly diagnosed NODAT. Patient recruitment and follow-up are conducted at the Medical University of Vienna. The study recruitment has started in February 2010.

### Study setting

**Figure 1:**
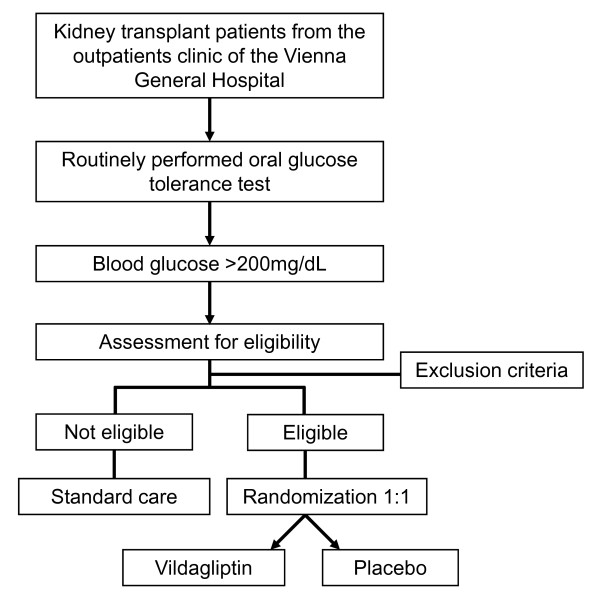
**Flowchart of the Study**.

**Figure 2:**
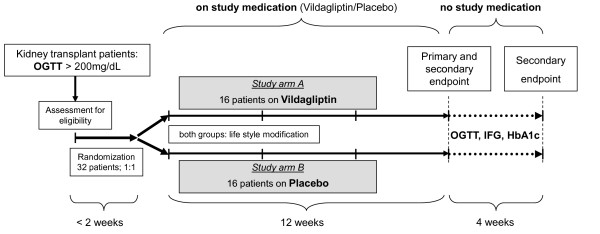
**Study procedures**. OGTT, oral glucose tolerance test.

Patients with a stable kidney allograft, more than 6 months after transplantation, without a history of T1DM or T2DM routinely undergo an OGTT at our outpatient department. All patients with a pathological OGTT (serum glucose levels ≥ 200 mg/d (11.1 mmol/L)) are classified as patients suffering from NODAT. 32 Patients eligible for the study are invited to the outpatient clinic and therapeutic options are discussed. Patients who are willing to take part in the study and have signed their informed consent form are randomized in a 1:1 ratio into study arm A (vildagliptin) or study arm B (placebo). The detailed study flow chart and an overview of study procedures are depicted in Figure 1 and 2, respectively.

### Study intervention

Patients will receive their study medication (vildagliptin or placebo) with instructions to take it once daily 30 minutes before breakfast. Patients will receive continuous counseling on lifestyle modification (e.g. diet, physical exercise) until the end of the study. Participants in the study have to be on a triple immunosuppressive therapy consisting of a CNI (tacrolimus or cyclosporine A), prednisolone, and mycophenolic acid, either as the prodrug (mycophenolate mofetil) or as delayed-release mycophenolic sodium. All changes in concomitant medication will be recorded. Patients will have a visit at our outpatient clinic during weeks 2, 4, 8, 12, and 16 (Figure 2). At each visit, blood samples are collected to determine blood parameters including complete blood count, serum chemistry, C-reactive protein, creatinine, calculated glomerular filtration rate (cGFR) using the "Modification of Diet in Renal Disease" (MDRD) formula, potassium, sodium, phosphate, chloride, calcium, total bilirubin, ALAT, ASAT, total protein, LDL, HDL, triglycerides. Patients with a cGFR between 30 and 50 mL/min./1.73 m^2 ^will have weekly blood checks comprising creatinine and ASAT/ALAT during the first month for safety reasons. Each patient is expected to parcipate in the study for 120 days. Unblinding of the study will be performed after the end of the complete trial. Patients whose OGTT did not improve 4 months after study start will be treated by the physicians of our outpatient clinic according to the guidelines.

### Informed consent

The investigator explains the nature of the study, its purpose, procedures, expected duration, and the potential risks and benefits associated with study participation along with any discomfort that may be expected. Patients will be informed about the strict confidentiality of their subject data, but also that their medical records may be reviewed for trial purposes by authorized individuals other than their treating physician. Each subject will be informed that study participation is voluntary and withdrawal is possible at any time during the study period. Withdrawal will not prejudice the subject's subsequent care. Subjects are given time to read and understand the statements before signing consent and dating the document. Subjects receive a copy of the signed written statement and the original copy of the informed consent is stored in the investigator study files. No subject is entered into the study until informed consent has been obtained.

### Safety assessments

Safety assessments will include the monitoring and recording of all adverse events (AE), including serious adverse events (SAE). An AE is any undesirable experience associated with the use of a medical product in a patient. An SAE is defined as any untoward medical occurrence that at any dose results in death, is life-threatening, requires inpatient hospitalization or prolongation of existing hospitalization, or results in persistent or significant disability/incapacity. The most probable AEs caused by vildagliptin are consistent with the known side-effects, which are the cause of the previously described exclusion criteria (table 1), such as wound healing disorders or severe renal impairment. The interruption or premature discontinuation of the study drugs might be triggered by AE, diagnostic or therapeutic procedures, abnormal laboratory values (e.g. basal ASAT/ALAT 50% elevated or more, serum creatinine 25% elevated or more) and for administrative reasons, in particular the withdrawal of the patient's consent.

**Table 1:** Patients inclusion and exclusion criteria of the study.

INCLUSION CRITERIA	EXCLUSION CRITERIA
≥18 years	Patients with prior **history of type 1 or type 2 diabetes**
	
Newly diagnosed **NODAT **defined by pathologic OGTT (2 h, 75 mg glucose): glucose ≥200 mg/dL	**Body mass index **(BMI) **> 40 kg/m^2^** - **Pregnancy**
	
**Renal transplantation **(deceased or living donor) and treatment with the standard immunosuppressionat our center, consisting of a triple therapy with tacrolimusor cyclosporine A, mycophenolatemofetil, and prednisone	**Severe renal impairment **(GFR < 30 mL/min./1.73 m^2^)
	
**Stable graft function **for more than 6 months post transplant	**Severe liver impairment **(ASAT/ALAT levels over threefold elevated compared to reference values)
	
**Informed consent **of the patient	Severe blood glucose elevation with the need for **insulin therapy **or

### Statistical analysis plan

The statistical analysis plan (SAP) provides full details regarding the analyses, the data display, and the algorithms to be used for data derivations. The SAP includes the definition of major and minor protocol deviations which will be identified by medically trained staff before the study closure. Safety and tolerability are analyzed descriptively. Safety analysis is performed on the ITT population.

The study sample will consist of 32 patients with newly diagnosed NODAT. For the primary endpoint analysis, we will assess the differences between treatment and control group in the 2 h glucose value obtained during an OGTT (75 g glucose) after 3 months of vildagliptin or placebo treatment. Based on a two-sided testing and a standard deviation of 20% in relative changes of 2 h OGTT glucose values, α = 0.05 and ß = 0.2, a sample size of 16 patients per group can detect a minimal difference in serum glucose level of 20 mg/dl at the 2 hour time point of the OGTT when comparing baseline levels to levels on day 90. The "Last observation carried forward" (LOCF) method will be used for missing data.

Two different analysis sets are defined for safety and efficacy, respectively. The efficacy of vildagliptin is assessed in all subjects who received the study drug (at least one dose) and did not violate the protocol in a way that might affect the evaluation of the effect of the study drug(s) on the primary objective, i.e. without major protocol violations. The per-protocol set is employed in the analysis of efficacy variables. A sensitivity analysis will be performed for efficacy with the ITT population.

The safety analysis set includes subjects who were randomized and received at least one dose of the study drug (modified intention to treat). The safety set is employed in the analysis of tolerability and safety variables. Statistical analysis is performed with SPSS.

### Approval of the ethics committee and the regulatory authority

The trial is performed in accordance with the Declaration of Helsinki as well as the Austrian drug law. It subscribes to the principles outlined in the most recent version of the International Conference on Harmonization on Good Clinical. Approvals were obtained from the ethics committee of the Medical University of Vienna and the Vienna General Hospital (Reference Number 645/2009) and from the Austrian regulatory authority (Federal Office for Safety in Health Care, Austrian Agency for Health and Food Safety) and was registered to the European Clinical Trials Database (EUDRACT number: 2009-14405-14). The study has also been registered in a public clinical trial database (Identifier Number NCT00980356, http://clinicaltrial.gov).

## Discussion

### Risk-benefit assessment

We expect all patients participating in this study to benefit because of patient counseling and emphasis placed on life-style modification in both study arms. Counseling is performed according to the guidelines of the International Diabetes Federation (IDF) [4]. If the hypothesis is true, the vildagliptin group (study arm A) will experience improved glycemic control. Vildagliptin is well tolerated in patients with mild to moderate renal impairment [20]. Patients with severe renal impairment (GFR < 30 mL/min/1.73 m^2^) will not be included in our study. Patients with a GFR between 30 and 50 mL/min./1.73 m^2 ^will have weekly visits at our outpatient clinic during the first 4 weeks (serum-creatinine and ASAT/ALAT) for safety. If renal function declines for any reason to a level below 30 mL/min/1.73 m^2^, administration of the study medication will be stopped.

NODAT continues to be a common and serious metabolic complication after organ transplantation. Currently, NODAT is diagnosed and treated like T2DM, but there is only limited evidence about the efficacy and safety of the novel antidiabetic drug vildagliptin in patients with NODAT, although it is already commonly used in T2DM. Based on the differences in pathophysiology between T2DM and NODAT, the complex drug profiles in transplanted patients, and the possible influence of renal impairment on the pharmacokinetic properties of vildagliptin, the antidiabetic efficiency of this drug in NODAT remains to be established. This trial will investigate whether vildagliptin is efficient and safe in patients with NODAT.

## Competing interests

This academic study is sponsored by the Medical University of Vienna, Austria. The authors do not receive any reimbursement or financial benefits and declare that they have no competing interests.

## Authors' contributions

MHa made substantial contributions to the conception and design of the study. He was involved in drafting the study protocol and wrote this manuscript. HCV, JW, MHe, WHH, DD, and TW participated in the design of the study and its coordination and helped to draft the manuscript. JP participates in the design of the study by giving advice on statistics and will be involved in the statistical analysis. GS will be in charge of the production, blinding and dispensing of study medication and helped to draft the manuscript. MDS is the principal investigator, responsible for recruitment and trial coordination. He developed the study idea and made substantial contributions to conception and design. Moreover, he was involved in drafting and revising the study protocol as well as this manuscript. All authors will participate in the implementation or analysis of this study and approved the final manuscript. All authors have read and approved this manuscript.
